# Robust training attenuates TBI-induced deficits in reference and working memory on the radial 8-arm maze

**DOI:** 10.3389/fnbeh.2013.00038

**Published:** 2013-05-03

**Authors:** Veronica Sebastian, Aissatou Diallo, Douglas S. F. Ling, Peter A. Serrano

**Affiliations:** ^1^Department of Psychology, Hunter CollegeNew York, NY, USA; ^2^Department of Physiology and Pharmacology, SUNY Downstate Medical CenterBrooklyn, NY, USA; ^3^The Robert F. Furchgott Center for Neural and Behavioral Science, SUNY Downstate Medical CenterBrooklyn, NY, USA; ^4^Department of Psychology, The Graduate Center of CUNYNew York, NY, USA

**Keywords:** traumatic brain injury, radial arm maze, working memory, reference memory, robust training

## Abstract

Globally, it is estimated that nearly 10 million people sustain severe brain injuries leading to hospitalization and/or death every year. Amongst survivors, traumatic brain injury (TBI) results in a wide variety of physical, emotional and cognitive deficits. The most common cognitive deficit associated with TBI is memory loss, involving impairments in spatial reference and working memory. However, the majority of research thus far has characterized the deficits associated with TBI on either reference or working memory systems separately, without investigating how they interact within a single task. Thus, we examined the effects of TBI on short-term working and long-term reference memory using the radial 8-arm maze (RAM) with a sequence of four baited and four unbaited arms. Subjects were given 10 daily trials for 6 days followed by a memory retrieval test 2 weeks after training. Multiple training trials not only provide robust training, but also test the subjects' ability to frequently update short-term memory while learning the reference rules of the task. Our results show that TBI significantly impaired short-term working memory function on previously acquired spatial information but has little effect on long-term reference memory. Additionally, TBI significantly increased working memory errors during acquisition and reference memory errors during retention testing 2 weeks later. With a longer recovery period after TBI, the robust RAM training mitigated the reference memory deficit in retention but not the short-term working memory deficit during acquisition. These results identify the resiliency and vulnerabilities of short-term working and long-term reference memory to TBI in the context of robust training. The data highlight the role of cognitive training and other behavioral remediation strategies implicated in attenuating deficits associated with TBI.

## Introduction

Traumatic brain injury (TBI), resulting in temporary or permanent impairment of cognitive abilities and physical functioning, is a major public health problem around the world (Langlois et al., 2004; Rutland-Brown et al., 2006). Globally, it is estimated that nearly 10 million people per year sustain severe brain injuries leading to hospitalization and/or death (Langlois et al., 2006). In the United States alone, it is estimated that over 1 million people are affected by TBI, resulting in 1.2 million emergency room visits, 290,000 hospitalizations and 51,000 deaths on an annual basis (Rutland-Brown et al., 2006). While TBI is fatal to many, individuals who manage to survive the initial trauma experience a host of long-term sequelae, ranging from subtle psychiatric symptoms to chronic physical and cognitive disabilities, such as alcoholism, stress, depression, epilepsy, and Alzheimer's disease (Dikmen et al., 2001; Fleminger et al., 2003; Langlois et al., 2004).

Although TBI can disrupt a multitude of brain functions, the most common cognitive deficit is memory loss, involving retrograde and/or anterograde amnesia, difficulty acquiring new information, and impairments in spatial reference and working memory (Hamm et al., 1996; Kobori and Dash, 2006; Whiting and Hamm, 2008; Hoskison et al., 2009; Atkins, 2011). Previous studies, however, have focused on characterizing TBI-associated deficits in reference or working memory systems independently, most commonly using the Morris water maze task (Smith et al., 1991; Dash et al., 1995; Bramlett et al., 1997; Scheff et al., 1997; Sanders et al., 1999; Griesbach et al., 2004). In order to identify whether the brain can compensate for deficits in one memory system with another, it is important to evaluate these systems in a single paradigm.

The radial 8-arm maze (RAM) is a particularly useful task with which to characterize TBI-induced deficits in both reference and working memory during learning and/or memory retrieval (Lyeth et al., 1990; Soblosky et al., 1996b). The paradigm challenges the rats to learn the long-term rules of the location of the baited arms (reference memory) while simultaneously remembering which arms have been visited (working memory). The RAM task, utilizing four baited and four unbaited arms, taxes the rats' ability to juggle both short-term working and long-term reference memory systems (Baddeley and Hitch, 1994; Abrahams et al., 1997). In addition to its ethological relevance to rodent foraging behaviors (Olton and Samuelson, 1976; Floresco et al., 1997), the RAM requires the activity of several brain regions impacted by TBI. In particular, lesion and electrophysiological studies have identified the importance of the hippocampus (Jarrard, 1978; Olton et al., 1979) and prefrontal cortex (Floresco et al., 1997) in completing the task. These regions are frequently damaged in rodent models of TBI (Chen et al., 2003; Hoskison et al., 2009; Ariza et al., 2006). Our particular RAM protocol has the added benefit of massed training trials over several days that yields a robust spatial memory. The effects of extensive training have been reported to mitigate learning and memory deficits across various paradigms (Wallace et al., 1980; Beatty et al., 1985; Stewart et al., 1989; Daumas et al., 2008; Zhang et al., 2011). Thus, we examine the degree to which overtraining on the RAM can produce a robust spatial memory capable of withstanding TBI and preserving memory function.

## Materials and methods

### Subjects

**Figure 1:**
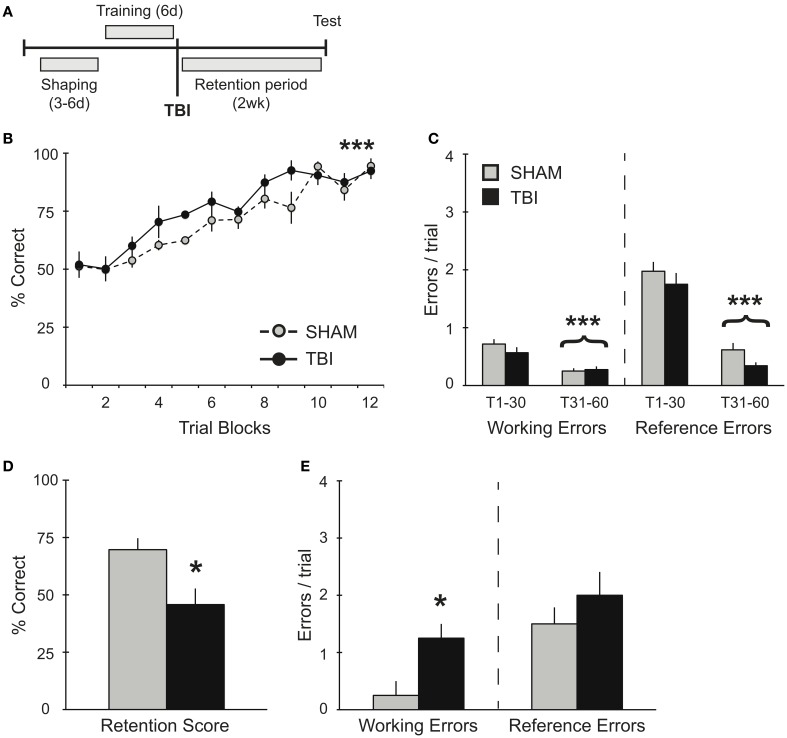
**TBI post-training induces working memory deficits during retention testing. (A)** Schematic diagram of the experimental design and timeline for experiment 1. **(B,C)** During training, there were no differences observed between groups during their pre-surgery assessment for acquisition of the RAM task. There was a significant improvement in percent correct and significant decreases in working and reference memory errors across training days for both groups (^***^p < 0.001). **(D,E)** During retention testing, TBI impaired memory retrieval with a decrease in percent correct score and an increase in working memory errors (^*^p < 0.05).

**Figure 2:**
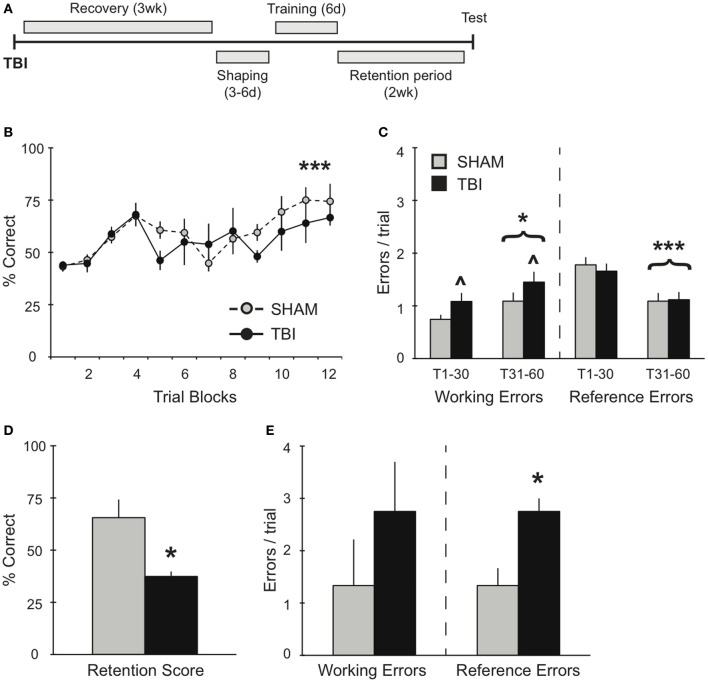
**TBI induces differential memory deficits during training and retention testing 3 weeks post-injury. (A)** Schematic diagram of the experimental design and timeline for experiment 2. **(B,C)** During training, there was a significant improvement in percent correct and a significant decrease in reference errors across training days for both groups (^***^p < 0.001). There was also an overall increase in working memory errors over time (^*^p < 0.05) and a deficit in working memory in the TBI group (^∧^*p* < 0.05). **(D,E)** During retention testing, TBI impaired memory retrieval with a decrease in percent correct score and an increase in reference memory errors (^*^p < 0.05).

**Figure 3:**
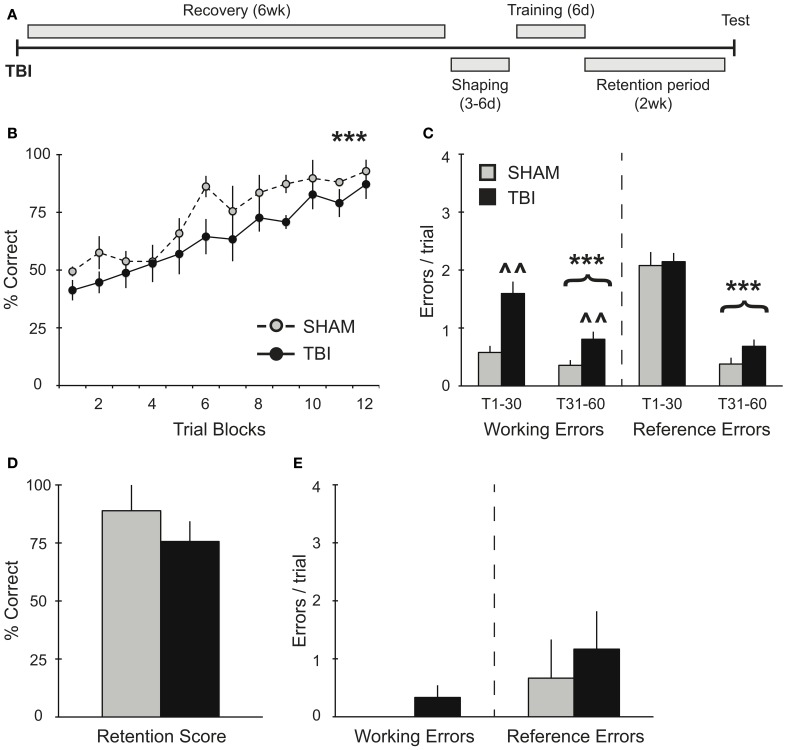
**TBI induces working memory deficits during training 6 weeks post-injury but does not impact memory retention. (A)** Schematic diagram of the experimental design and timeline for experiment 3. **(B,C)** During training, there was a significant improvement in percent correct and significant decreases in working and reference memory errors across training days for both groups (^***^p < 0.001). There was also a deficit in working memory in the TBI group (^∧∧^*p* < 0.01). **(D,E)** During retention testing, there were no significant differences in performance between groups.

Adult male Sprague-Dawley rats (Charles River; Boston, MA) weighing 270–300 g were used for the current experiment. Rats were individually housed in plastic cages (48 × 27 × 16 cm) containing hardwood bedding. Animal quarters were maintained at constant temperature (22 ± 1°C) and relative humidity (40–50%) with a 12 h light/dark cycle (lights on at 8 AM). Food (Harlan Teklad; Frederick, MD) and water were available *ad libitum* prior to behavioral training and during the recovery period. Subjects were divided into three cohorts (each with TBI and Sham controls) for three separate experiments (see Figures 1A, 2A, 3A). All procedures were performed in accordance with the NIH Guide for the Care and Use of Laboratory Animals and approved by the Institutional Animal Care and Use Committee at Hunter College.

### Behavioral procedure

Rats were trained on an eight-arm radial arm maze as previously described (Schrott et al., 2008; Serrano et al., 2008). Briefly, rats were shaped for 3–6 days prior to training, during which time they were food-restricted to gradually reach 85% of their free-feeding weight. During this period, rats were also habituated to the maze and to the sweetened oatmeal mash (Maypo; International Home Foods) that served as a food reward, using three-10 min trials per day. For each shaping trial, rats were released individually in the center of the maze and allowed 10 min to forage and collect food from all arms. After shaping, each rat was randomly assigned four arms to be baited for the remainder of the experiment. To prevent the use of internal cues, the maze was rotated 90° daily while the spatial location of the baited arms with respect to the room cues remained constant. During training, the assigned arms were each baited with 0.13 g of oatmeal placed at the end of each arm and the other remaining four arms were unbaited. The sequence of baited/unbaited arms remained constant throughout the experiment for each subject. Rats were trained for a period of 6 days with 10 consecutive trials per day (60 trials total). During training, rats were placed onto the center of the maze and confined with a black box (20 × 20 × 20 cm) prior to the beginning of each trial. Once released, the rat remained on the maze until it collected food from all baited arms or until 3 min had elapsed. The sequence of arms entered and the latency to find all four food rewards was recorded. A percent correct score and a tally of total errors were calculated for every trial. Rats made two types of errors: reference memory errors (entries into unbaited arms) and working memory errors (repeated entries into previously explored arms). Two weeks after the last training trial, subjects were tested for memory retention with 3–5 additional trials on the maze. Only the median trial was selected for analysis to control for a learning effect over the additional trials.

### Controlled cortical impact (CCI) surgery

Rats were anesthetized with pentobarbital (45 mg/kg, i.p.) and then placed on a stereotaxic frame. A midline incision was made to expose the skull. Animals were subjected to a 6 mm diameter craniotomy centered over the right somatosensory cortex, equidistant from bregma and lambda. Craniotomy was carefully performed to ensure that the dura remained intact. Controlled cortical impact (CCI) injury was induced using a cortical impact device (myNeurolab; St. Louis, MO) with a pneumatic steel impactor tip (5 mm diameter) angled at 22° from the sagittal plane and perpendicular to the site of injury (Yang et al., 2010). The impactor tip penetrated the exposed cortex to a depth of 2 mm with a velocity of 4 m/s. After injury, the wound was gently compressed to stop bleeding and cleaned with sterile saline before being covered with a plastic cap over the skull. Sham operated controls underwent the same craniotomy procedure but did not receive the impact. Rats were fully ambulatory within 60 min after surgery and no mortalities occurred from this procedure. All subjects received fluids (single dose) and pain medication (5 mg/kg ketofen for 3 days), and were closely monitored for 6 h post-surgery. Both groups continued to be monitored daily until the end of each experiment.

### Statistical analyses

Performance on the radial arm maze during the training period was analyzed using two-way, repeated measure ANOVAs. Learning curves for percent correct were analyzed as trial blocks (five trials each, two trial blocks/day). Performance on the radial arm maze during the retention test was analyzed by using unequal variance, one-tailed *t*-test.

## Results

In order to assess the effects of TBI on spatial memory retrieval in the RAM task (Figure 1A), groups of rats were trained on the task for 60 trials (10 trials/day). This training paradigm results in a long-term spatial memory that is reflected in several days of asymptotic performance (Trial Blocks 8–12). As expected, prior to surgery, both groups acquired the RAM task equivalently, showing significant increases in percent correct scores [Figure 1B, RM ANOVA *F*_(1, 66)_ = 27.83, *p* < 0.001, *n* = 4 per condition] and decreases in both working [*F*_(1, 46)_ = 24.55, *p* < 0.001] and reference [*F*_(1, 46)_ = 144.5, *p* < 0.001] memory errors during the second half of training compared to the first half (Trials 1–30 vs. Trials 31–60, Figure 1C). While both groups showed no differences in their acquisition of the task, 2 weeks after TBI or Sham surgery, memory retention for the RAM task was significantly impaired in the TBI group (Figure 1D). Specifically, TBI increased the number of working memory errors, but left reference memory intact (Figure 1E).

To further evaluate the effects of TBI on spatial working memory, experiment 2 examined the effects of TBI on the acquisition of the RAM task 3 weeks after injury (Figure 2A). Both groups demonstrated significant improvements over days of training [Figure 2B, RM ANOVA *F*_(1, 55)_ = 4.735, *p* < 0.001, *n* = 3–4 per condition], in addition to a decrease in reference memory errors during the second half of training [Figure 2C, *F*_(1, 40)_ = 27.84, *p* < 0.001]. Although there were no differences in percent correct between Sham and TBI groups, overall they performed worse than subjects in experiment 1, which were trained prior to surgery [*F*_(1, 143)_ = 36.69, *p* < 0.0001]. In addition, both groups made significantly more working memory errors (Figure 2C) during the second half of training [*F*_(1, 40)_ = 4.191, *p* < 0.05], while the TBI group overall made significantly more working memory errors compared to Shams throughout the training [*F*_(1, 40)_ = 4.633, *p* < 0.05]. The difference in memory acquisition between groups was also reflected in their retention test 2 weeks later: the TBI group had a significantly lower percent correct score compared to Shams (Figure 2D). There were no differences in their working memory errors, but a significant increase in the TBI group for reference memory errors (Figure 2E).

The effect of additional recovery time after injury was assessed in experiment 3 (Figure 3A). Groups of rats were trained on the RAM task 6 weeks after TBI or Sham surgery. As in previous experiments, both groups showed a significant improvement in their percent correct scores during acquisition over time [Figure 3B, RM ANOVA *F*_(1, 77)_ = 19.34, *p* < 0.001, *n* = 3–6 per condition] without any differences between TBI and Sham groups. Overall, subjects in experiment 3 did not perform significantly different from subjects in experiment 1, which were trained prior to surgery [*F*_(1, 165)_ = 3.856, *p* = 0.068]. Again, both TBI and Sham groups showed significant reductions in working [*F*_(1, 52)_ = 14.77, *p* < 0.001] and reference [*F*_(1, 52)_ = 208.9, *p* < 0.001] memory errors during the second half of training compared to the first half (Figure 3C). However, the TBI group made significantly more working memory errors throughout the training compared to Shams [*F*_(1, 40)_ = 11.09, *p* < 0.01]. During retention testing 2 weeks after training, there were no differences in retention between conditions (Figures 3D,E).

## Discussion

The effects of TBI on rodent spatial reference and working memory have significant translational relevance (McAllister et al., 2001), in that they realistically model memory deficits in human subjects (Ramos et al., 2003). While many of these deficits are long lasting, it appears that the cellular and molecular mechanisms underlying working memory dysfunction change over time (Gong et al., 1999; Hu et al., 2004; Hoskison et al., 2009), suggesting that there are corresponding changes in memory function that occur with longer recovery periods. In the present study, we explore this phenomenon by characterizing the learning and memory components that are at risk after injury, either during retention alone or during acquisition *and* retention, using a robust training protocol to examine both reference and working memory systems. Reference memory is associated with long-term memory for the task rules. In the case of the RAM, it refers to the stationary location of the baited arms, which remains constant across all trials. Additionally, during each training trial, rats are required to take into account which of the eight arms they have already visited. This skill, referred to as working memory (Honig, 1978; Olton et al., 1979), must be maintained until all the food rewards are retrieved in a single trial. At the start of every new trial, the working memory load must be reset in order to successfully complete the task.

We find that TBI affects memory retrieval for spatial information acquired prior to injury and that these deficits in memory retrieval are specific to short-term working, but not long-term reference, memory. Further, we find that shortly (3 weeks) after TBI, the acquisition of new spatial information is impaired. We identify deficits in short-term working memory during training and in long-term reference memory during retention testing 2 weeks post-training. Similarly, with a longer recovery period, we find that deficits in short-term working memory continue to be prevalent during training. However, these subjects show no deficit on the long-term memory retention at 2 weeks. These data suggest that some of the long-term memory deficits associated with TBI can be remediated with longer recovery time and robust training, but lingering deficits in short-term working memory remain. Addressed here, we explore the functional significance of a short-term working memory deficit and raise the question as to other potential remediation factors applied to improving memory deficits associated with TBI.

### Robust spatial training compensates for learning and memory deficits

Several studies have shown a reversal of short-term memory deficits in overtrained transgenic mice and an elimination of the spatial memory deficits with subsequent re-training (Daumas et al., 2008; Zhang et al., 2011). Additional evidence has demonstrated that overtraining or training to criteria minimizes memory deficits in older animals (Wallace et al., 1980; Beatty et al., 1985; Stewart et al., 1989). Similarly, we use a robust training protocol on the RAM that is known to result in successful memory retrieval up to 1 month post-training (unpublished data) and thus, to dramatically increase the ability to retain spatial information. Our data in experiment 1 (Figures 1B,C) illustrates the rate of acquisition and the error profile involving working and reference memory in uninjured subjects (prior to surgery). In spite of this robust training paradigm, TBI significantly impaired working memory during retention testing 2 weeks post-training (Figures 1D,E). These findings are consistent with other reports in rodents (Zohar et al., 2003; Djebaili et al., 2004; Xia et al., 2012), and humans (Vallat-Azouvi et al., 2007; Willmott et al., 2009), all identifying similar patterns of deficits for previously acquired spatial information.

In experiments 2 and 3, TBI groups demonstrated no deficits in overall percent correct during the acquisition phase compared to Shams (Figures 2, 3). It is worth noting that, although there was no difference in percent correct score between TBI and Sham groups during training for experiment 2, overall these subjects performed worse than subjects in experiment 1, which were trained prior to surgery (Figures 1B, 2B). Particularly in the case of the Sham group, this effect is most probably the result of the craniotomy procedure, which can also induce a distinct inflammatory response and affect motor function briefly post-surgery (Cole et al., 2011; Lagraoui et al., 2012). Nonetheless, compared to Shams, TBI subjects in experiments 2 and 3 still show increases in working memory errors throughout this period and, in the case of experiment 2, TBI subjects failed to perform on the retention test (Figures 2D,E). These results indicate that our robust training protocol of massed trials over several days is sensitive enough to identify differences in spatial memory performance affected by recovery period after TBI. Further experiments are needed using weaker training protocol to determine the degree to which training has a direct effect maintaining memory.

### Selective memory deficits on RAM acquisition and retention

Since many previous studies on the effects of TBI on memory specifically chose a working memory paradigm to examine, their results are limited to this one particular memory function. In our application of the RAM, we were able to assess both reference and working memory systems. The dissociation between reference and working memories is more difficult to accomplish with other tasks, which can be adapted using protocols for either memory system, but not both simultaneously (Yoshihara and Ichitani, 2004). In an intact subject, our training protocol produced a prominent decline in reference memory errors between trials 1–30 and 31–60, the first and second halves of training (Figure 1C). While working memory errors also significantly decreased, the average number of errors was lower compared to reference memory errors. This suggests that animals with this acquisition profile have little difficulty in acquiring the working memory component of this RAM protocol. Certainly, what difficulty remains is not with remembering which food pellets have been collected during a single, 3-min trial, since rats can maintain a working memory buffer up to 2 h (Yoshihara and Ichitani, 2004). Thus, the challenge to the working memory resides in balancing working memory load while learning or recalling the reference memory rules simultaneously, an effect that is greatly exacerbated by TBI. Training rats to criterion for 5 consecutive days using a single trial per day on the exclusively working memory version of the RAM (all eight arms baited) was reported to take approximately 10 days. With this protocol, TBI had no effect on working memory (Soblosky et al., 1996b). This comparison suggests that the additional memory load in our paradigm may come from having to clear the previous trial's working memory information repeatedly within a span of 30–40 min, the time it takes to complete one day's training session. Therefore, we interpret the prominent working memory deficit during retrieval as the inability to reset working memory load. Interestingly, the deficits that we identify in updating short-term working memory are consistent with the type of deficit found in aging animals, where working memory deficits become more pronounced as the number of previously reinforced choices and locations to be remembered is increased (Bimonte et al., 2003).

In experiment 2, animals had a limited time to recover before beginning RAM training. Though they showed significant improvement in their overall scores (Figure 2B), both groups failed to show any improvement in working memory errors between the first and second half of training (Figure 2C). In addition, the TBI group made greater working memory errors during training and performed significantly worse in the retention test compared to Sham controls (Figures 2C–E). We believe that this deficiency in working memory function during training is directly responsible for the deficit in long-term memory retention. Without the ability to accurately perform short-term working memory function, the long-term consolidation is undermined and retrieval is negatively affected 2 weeks later. Our data highlight how successful RAM acquisition and retention requires significant improvements in both working and reference errors for long-term memory retrieval function to be preserved. In a similar set-up for the RAM, where both reference and working memory errors were assessed after TBI, an increase in reference but not working memory errors was reported (Soblosky et al., 1996b). The inconsistency of our data with this result could be due to differences in training protocols. We used a massed training protocol that allows for memory retention 1 month post-training (unpublished data) and that typically has asymptotic performance for at least 20 trials, whereas Soblosky et al. only trained to five trials of asymptotic performance. Secondly, the injury induced by Soblosky et al. was much more severe than our injury paradigm (Yang et al., 2010), both in terms of impact velocity and area of cortex damaged. Such a severe injury could result in more serious deficits in reference memory, which is associated with long-term memory for the rules of the radial arm maze. Furthermore, when reference memory is compromised on the four baited arms version of the RAM, there may not be sufficient remaining choices to allow for identification of short-term working memory errors.

In experiment 3, where subjects had 6 weeks to recover, we show that memory retrieval remained intact after injury (Figures 3D,E). We find that both groups were able to significantly improve working memory performance during the second half of training, though TBI subjects made more working memory errors overall. We interpret this as a practice effect that allows them to compensate for the TBI specific affects on updating short-term working memory. The ability to compensate for some of the working-memory deficits on long-term memory retrieval are consistent with studies showing that is takes 4–5 weeks for rodents to recover behavioral function (Soblosky et al., 1996a; Piot-Grosjean et al., 2001). However, others have shown persistent cognitive deficits lasting several months post-injury (Lindner et al., 1998), suggesting that the improved performance of subjects in experiment 3 compared to experiment 2 was the cumulative result of both longer recovery times and robust training.

### Is the TBI behavioral deficit associated with a particular brain region?

The CCI model we use is known to affect several different brain regions including parietal and medial frontal cortices involved in memory and spatial localization (Dixon et al., 1991; Chen et al., 2003; Yang et al., 2010). The correlation with the impact and severity of the injury with recovery of function is well documented (Goodman et al., 1994; Markgraf et al., 2001). While many of these studies investigate the recovery effects on behavior over a much shorter time-window, here our investigation of a long-term recovery time-window extends the current understanding of recovery of behavioral function. Our data are consistent with findings that pre-frontal area lesions affect RAM working errors (Joel et al., 1997) and also reference memory errors (Kolb et al., 1982). Subjects with longer recovery times also faired better on acquiring the RAM task, which is consistent with a smaller lesion and a memory deficit restricted to working memory (Joel et al., 1997). Indeed, we show that subjects from experiment 2 given a shorter recovery period show both working memory deficits during training, and reference memory deficits during retention testing.

### Importance of overtraining and other behavioral remediation strategies after TBI

Much effort has been applied to finding the deficits associated with TBI in the hope that better treatment options will be developed. Overall, it appears that the injury itself requires several weeks to reach a stable recovery state (Soblosky et al., 1996a). Moreover, the chemistry and cellular morphology associated with the deficits change remarkably during the acute recovery phase, while the stable recovery phase reveals changes in spine morphology (Newcomb et al., 1999; Chen et al., 2003; Hoskison et al., 2009). Both of these findings are relevant to the data we present here, which identify the changing nature of the deficit but highlight the ability for the brain to compensate for the deficits with experience. Our work is consistent with several other studies where practice has improved performance on cognitive tasks, such as executive functioning and memory in military personnel (Bogdanova and Verfaellie, 2012). Additionally, both diet and exercise has been proven to be an effective remediation technique following TBI (Griesbach et al., 2009; Wu et al., 2011; Ying et al., 2012; Tyagi et al., 2013). Increased exercise in rodents within 2 days after injury improved cognitive deficits associated with object recognition memory (Chen et al., 2012) while an exercise regimen 3 months after injury improved working memory (Piao et al., 2013). Similarly, environmental enrichment has also been shown to improve both recovery and cognition in male and female rats (Matter et al., 2011; Cheng et al., 2012; Monaco et al., 2013). Together, these intervention strategies all cast a light on the importance of alternative behavioral therapies for improving the long-lasting effects of TBI on memory.

### Conflict of interest statement

The authors declare that the research was conducted in the absence of any commercial or financial relationships that could be construed as a potential conflict of interest.
